# Striking Dependence of Protein Sweetness on Water Quality: The Role of the Ionic Strength

**DOI:** 10.3389/fmolb.2021.705102

**Published:** 2021-07-22

**Authors:** Masoud Delfi, Alessandro Emendato, Piero Andrea Temussi, Delia Picone

**Affiliations:** ^1^ Department of Chemical Sciences, University of Naples “Federico II”, Complesso Universitario Monte S. Angelo, Naples, Italy; ^2^ UK Dementia Research Institute at King’s College London, The Maurice Wohl Institute, London, United Kingdom

**Keywords:** sweet proteins, wedge model, MNEI, thaumatin, sweet receptor, single chain monellin mutants

## Abstract

Sweet proteins are the sweetest natural molecules. This aspect prompted several proposals for their use as food additives, mainly because the amounts to be added to food would be very small and safe for people suffering from sucrose-linked diseases. During studies of sweet proteins as food additives we found that their sweetness is affected by water salinity, while there is no influence on protein’s structure. Parallel tasting of small size sweeteners revealed no influence of the water quality. This result is explained by the interference of ionic strength with the mechanism of action of sweet proteins and provides an experimental validation of the wedge model for the interaction of proteins with the sweet receptor.

## Introduction

Sweetness is elicited by a very large variety of molecules, both natural and synthetic (Temussi, 2006a; Temussi, 2007). Sweet proteins stand out not only because they have much larger molecular weight (and corresponding large volumes) with respect to all other sweeteners, but also, they are the sweetest known natural molecules up to date. Among all sweet proteins, Thaumatin (van der Wel and Loeve, 1972) and Monellin (Morris and Cagan, 1972) are the sweetest, being approximately 100,000 times sweeter than sucrose, on molar basis (Temussi, 2006b). Thaumatin is extracted from *Thaumatococcus danielli*, a West African plant, as a combination of two proteins: Thaumatin I and II (van der Wel and Loeve, 1972). Thaumatin is very soluble in water, up to 600 mg/ml, and is endowed by high thermal stability under acidic conditions, with retained sweetness at 80°C for several hours (Kaneko and Kitabatake, 2001). Monellin is another intensely sweet protein, isolated from *Dioscoreophyllum cumminsii*, a West Africa tropical plant (Inglett and May 1969). It is a small globular protein (94 residues), composed of two polypeptide chains. In Monellin, two peptide chains are linked together by non-covalent interactions forming a five-strand β-sheet half-wrapped around an α-helix (Ogata et al., 1987). In fact, Monellin undergoes irreversible denaturation and subsequently loss of sweetness when heated over 50°C due to the dissociation of the polypeptide chains (Kim et al., 1989). To enhance its thermal stability, a protein dubbed MNEI was designed by joining both subunits of Monellin through a Gly Phe linker (Tancredi et al., 1992). MNEI and others single chain Monellin variants reach temperatures higher than 70°C without loss of sweetness power (Kim et al., 1989).

It is well known that one receptor is sufficient to account for the taste of all sweet molecules, ranging from low molecular weight compounds to sweet proteins. This is a class C G-protein-coupled receptor (GPCR) formed by two similar polypeptides, T1R2 and T1R3 (Chandrashekar et al., 2006). From the knowledge of many other class C GPCRs it was clear that most low molecular weight sweet molecules bind to the orthosteric sites of the external domain of the receptor, the so-called Venus Flytrap domain (VFTD), but it was more difficult to explain the mechanism of interaction between the receptor and sweet proteins, whose sizes are typically two orders of magnitude bigger. Therefore, it was not possible to propose that sweet proteins bind to the same orthosteric sites that host small ligands. Among the different hypotheses formulated to explain the binding between sweet proteins and their receptor (Assadi-Porter et al., 2010; Jiang et al., 2004; Masuda et al., 2013; Temussi, 2002), the most credited one able to explain the behavior of all sweet proteins is called “wedge model” (Temussi, 2002). Its origin rests on the properties of a glutamate receptor, mGluR1, that was used originally to build the homology model of the sweet receptor (Kunishima et al., 2000). The crystallographers found that mGluR1 exists as an equilibrium mixture between an active and an inactive form, even in the absence of any ligand. Owing to the similarity between the glutamate receptor and the sweet receptor, it was also assumed that the last one existed as an equilibrium mixture between active and inactive forms. The equilibrium can be shifted in favor of the active conformation by the binding of low molecular weight sweet compounds within the orthosteric sites, but also by preferential external binding of a sweet protein to the active form (Temussi et al., 2002).

Considering the promising industrial applications of sweet proteins, some attempts have already been performed to improve the properties of MNEI based on the surface interactions with the sweet taste receptor predicted by the wedge model. Different MNEI mutants with improved sweetness and stability were successfully obtained through this approach. Among these one, dubbed Mut3, is characterized by the highest sweetness, quantified by a strong decrease of the sweetness threshold (from 1.64 mg/L for MNEI to 0.28 mg/L for Mut3) (Leone et al., 2016; Rega et al., 2015); a second one, called Mut9, showed an extraordinary thermal stability and also resistance upon boiling at pH 2.5 and 6.8, together with a two-fold improvement in the sweetness power (Delfi et al., 2021).

The exceptional sweetness of these proteins has led to the proposal of their use as sweeteners in food. In this framework, while investigating several practical aspects of the applicability of sweet proteins as food additives, we found that the sweetness was affected by the type of water used to dissolve the proteins and prepare the food products. In the present paper, we examined the influence of water quality on sweetness in a systematic way. We used 4 sweet proteins with different size and net charge, i.e., Thaumatin, MNEI, Mut3, and Mut9, to prepare drinking samples in 4 types of commercial waters with different ion contents. To understand the origin of this puzzling phenomenon we investigated first the possible influence on the conformation of sweet proteins and found that water quality has no influence whatsoever on protein conformation. At the same time, we determined beyond doubt that water does not change sweetness perception of small molecular weight sweeteners. We finally resorted to known mechanisms of sweetness for sweet proteins and found a simple explanation of the water effect based on the changes in waters ionic strength, thus providing an experimental validation of the wedge model of the interaction of sweet proteins with their receptor.

## Materials and Methods

### Materials

MNEI, Mut3 and Mut9 were obtained as previously described (Leone et al., 2016; Delfi et al., 2021), while Thaumatin was purchased from Sigma (Sigma-Aldrich, United States). Sucrose and sucralose were purchased from the markets. Protein concentration was assessed by UV absorbance at 280 nm using an extinction coefficient (at 0.1%) of 1.41 for MNEI and Mut9, 1.29 for Mut3, and 1.33 for Thaumatin. The water analyzed were HiPerSolv CHROMANORM HPLC grade (VWR), henceforth synthetically indicated as “HPLC”, and commercial mineral waters (Sant’Anna, Rocchetta, and Lieve), selected based on the mineral residue amounts, conductivity, and pH, as indicated in Supplementary Table 1.

### Sensory Analysis

The drinking samples were prepared with MNEI, Mut3, Mut9, and Thaumatin, in the four waters. MNEI, Mut3, Mut9, and Thaumatin samples were prepared with the concentrations of 2, 4, 6, 8, and 10 mg/L, while for Mut3 also lower concentrations (0.5 and 1 mg/L) were prepared due to its very high sweetness (Leone et al., 2016). As a further control, MNEI and Thaumatin were dissolved at a 10 mg/L concentration in HPLC water containing a NaCl concentration calculated to reproduce the ionic strength of the three mineral waters, according to the data reported on the labels (see Supplementary Table 1). A group of 5 panellists (2 males and 3 women, selected between students and researchers of the Department of Chemical Sciences), participated in the blind tasting sessions, carried out as described elsewhere (Leone at al., 2016). Two paper cups, one containing 5 ml of protein sample and the other 5 ml of commercial water were provided for the tasting and recording their evaluation from 0 to 5, as follows: 0: (no taste), 1: unidentified taste, 2: slightly sweet, 3: sweet, 4: very sweet, and 5: extremely sweet.

Samples of 40 g/L sucrose and 67 mg/L sucralose in the same types of water were prepared. The subjects tasted the sample solutions without any time constrains followed by spitting it out and rinsing their mouth thoroughly with mineral water within 1 min interval.

### Circular Dichroism Spectroscopy

CD measurements were performed on a Jasco J-1500 spectropolarimeter (Jasco, Essex, United Kingdom), equipped with a Peltier temperature control system (CTU-100), using a 1.0 cm quartz cell. The spectra measured in the far UV-range 195–250 nm (50 nm/min scan speed), and each experiment performed with 3 accumulations. Molar ellipticity per mean residue [θ] was calculated according to the formula:

$$\left[\theta \right]={\left[\theta \right]}_{\mathrm{obs}}\mathrm{mrw}/\left(\mathrm{10}\times 1\times C\right),\deg cm^{2}\mathrm{dmo}l^{-1}$$ (1)

Where [θ]_obs_ is the raw ellipticity values measured in degrees, mrw is the mean residue molecular weight of each protein (Da), C is the protein concentration in g/mL and l is the optical path length in cm.

### NMR Spectroscopy

^1^H NMR spectra were recorded at the University of Naples “Federico II” using a Bruker AVANCE 700 MHz spectrometer equipped with a cryo-probe, using excitation sculpting water suppression pulse sequence on resonance with the water signal. The protein samples were prepared in 90% commercial water (as indicated in the captions) and 10% D_2_O at the concentration of 3 mg/mL for MNEI and 6 mg/mL for Thaumatin, respectively. Spectra were acquired at 25 °C, with 64k fid points, 128 scans, 2 s recycle delay.

## Results

Four mineral waters belonging to two categories were selected for our study: HPLC and Sant’Anna, characterized by low mineral residues; Rocchetta and Lieve, having high content of mineral residues, as reported in Supplementary Table 1. A sensory analysis was performed on all the protein samples, showing in all the examined cases a clear dose-response effect. In addition, the secondary structures of all the proteins in the four different waters were examined by CD spectroscopy at the highest protein concentrations used in the tests (10 mg/L). Furthermore, a deeper comparison *via* ^1^H-NMR analysis was performed on selected combinations of proteins and water types, as detailed below.

### MNEI and its Mutants

**FIGURE 1:**
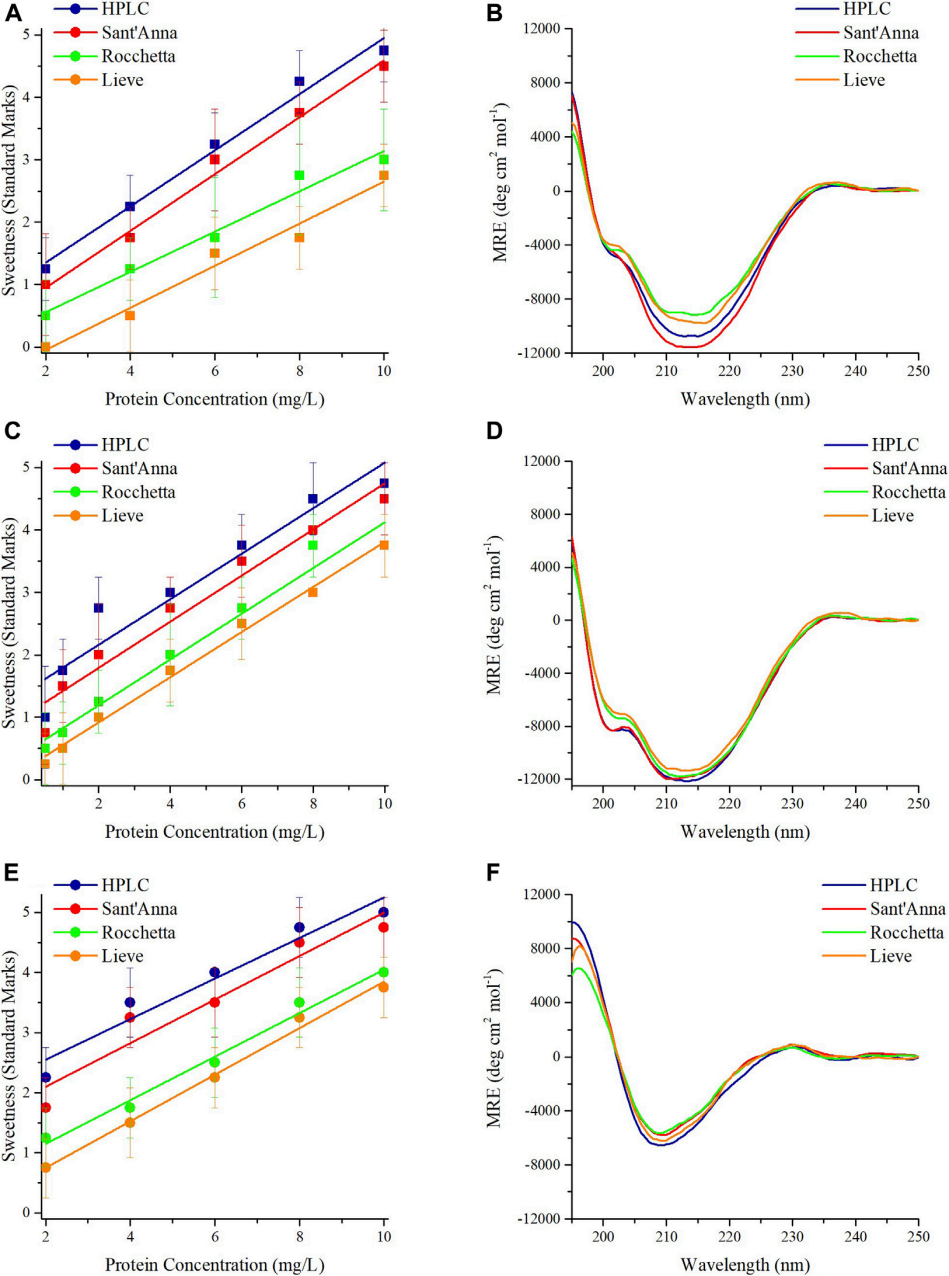
Sensory analysis and CD spectra of sweet proteins MNEI, Mut3, Mut9. Sensory analysis of MNEI **(A)**, Mut3 **(C)**, and Mut9 (**E**), at different concentrations in 4 commercial waters: blue) HPLC, red) Sant’Anna, green) Rocchetta, and orange) Lieve. CD spectra of MNEI **(B)**, Mut3 **(D)**, and Mut9 **(F)**, at 10 mg/L in 4 commercial waters.

Interestingly, MNEI and its mutants demonstrated almost identical behavior in terms of sensory analysis and secondary structures. The sweetness of proteins at 2 mg/L in HPLC and Sant’Anna was evaluated between the levels of unidentified taste and sweet. At higher protein concentrations, the sweetness intensity of MNEI, Mut3, and Mut9 increased up to a maximum for 10 mg/L, where it reached a value between very sweet and extremely sweet (Figures 1A,C,E). Samples prepared with Rocchetta and Lieve tasted less sweet at all concentrations. In fact, the samples at 2 mg/L protein concentration were tasteless (Lieve) or barely sweet (Rocchetta) for MNEI and its mutants. Upon increasing the protein concentration to 10 mg/L, the sweetness intensity of MNEI, Mut3, and Mut9 was assessed between the rates of slightly sweet to very sweet (Figures 1A,C,E).

The CD spectra of MNEI, Mut3 and Mut9 with the protein concentration of 10 mg/L were also analyzed. The structural architectures of MNEI, Mut3, and Mut9 reflected by CD spectra in all 4 types of commercial water are very similar (Figures 1B,D,F). The spectra were analyzed by deconvolution using the BestSel online tool (Micsonai et al., 2018), yielding variations of the secondary structures (Supplementary Table 2) far smaller than intrinsic errors in the deconvolution procedure (Nagy and Grubmüller, 2020).

### Thaumatin

**FIGURE 2:**
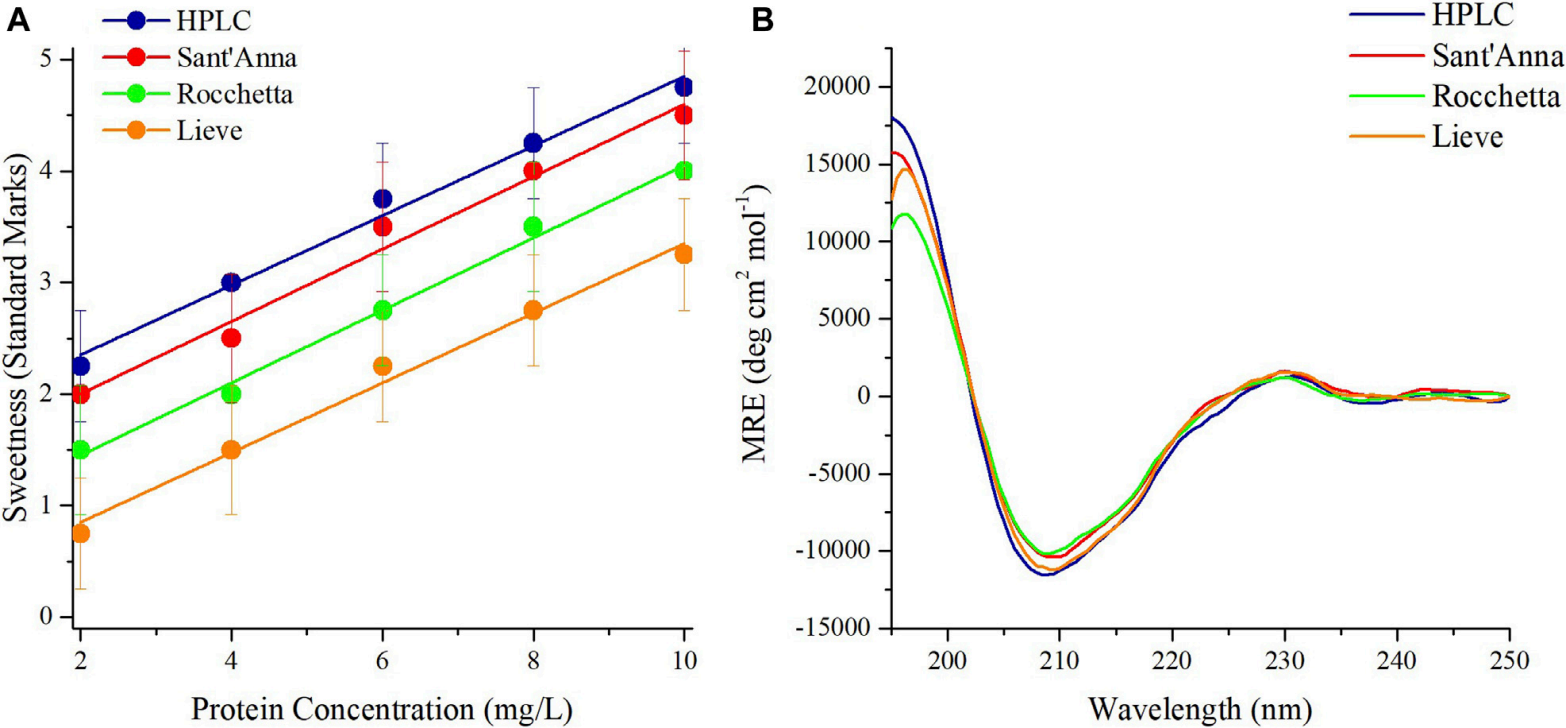
Sensory analysis and CD spectra of sweet protein Thaumatin. Sensory analysis of Thaumatin at different concentrations in 4 commercial waters: blue) HPLC, red) Sant’Anna, green) Rocchetta, and orange) Lieve **(A)**. CD spectra of Thaumatin at 10 mg/L in 4 commercial waters (**B**).

A sensory analysis was performed on Thaumatin using the same four commercial water types with protein concentrations ranging from 2 to 10 mg/L as well. At the lowest protein concentration, only Thaumatin prepared in HPLC and Sant’Anna tasted slightly sweet and the other two samples were almost tasteless (Figure 2A). The sweetness potency of 10 mg/L Thaumatin in HPLC and Sant’Anna was evaluated between the levels of very sweet and extremely sweet, while in Rocchetta and Lieve was perceived one level less sweet (Figure 2A). CD spectroscopy revealed that the spectra of Thaumatin in all types of water were highly superimposable (Figure 2B). The minor variations observed in the secondary structure contents (Supplementary Table 3) are again very small.

**FIGURE 3:**
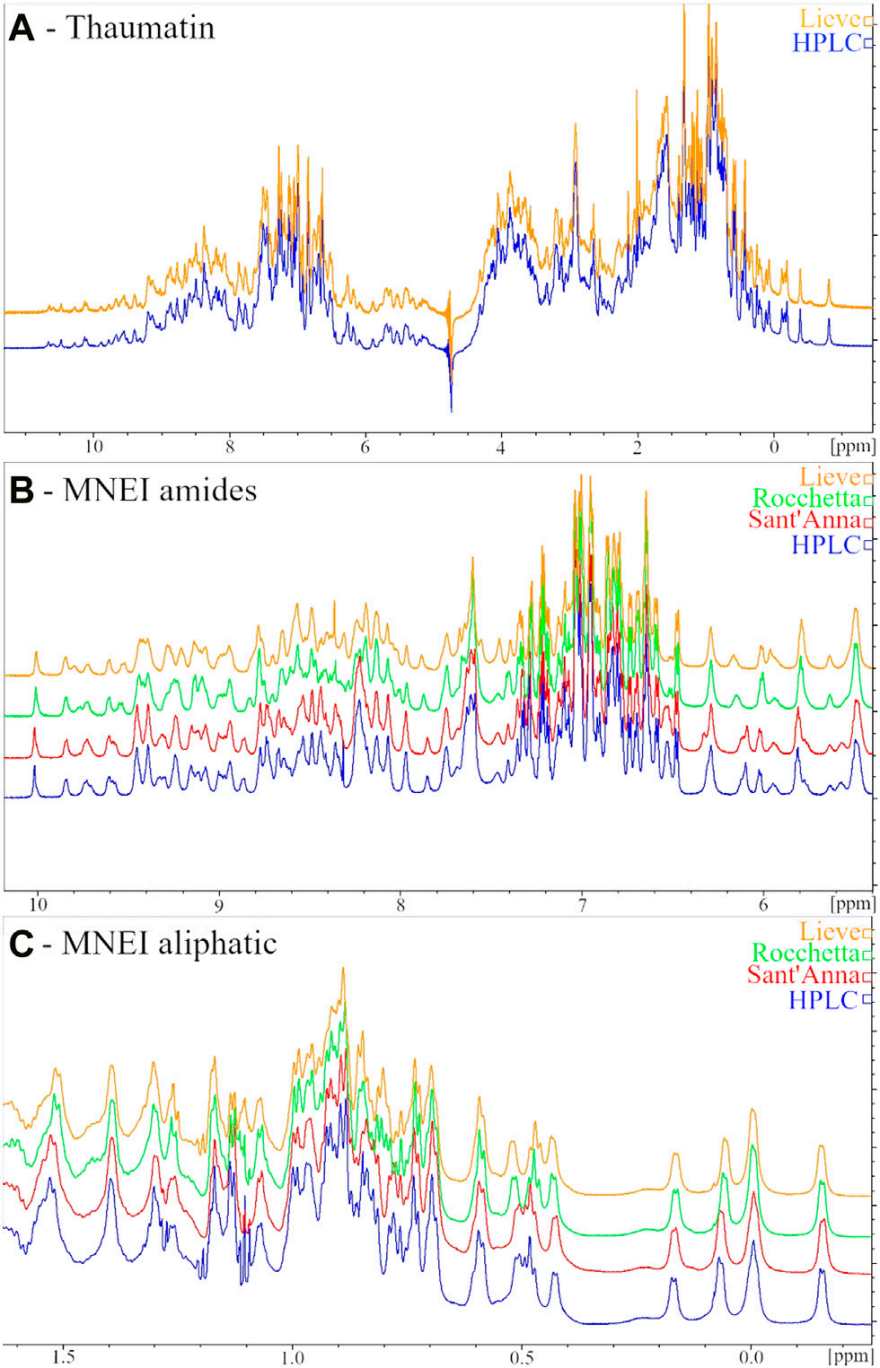
^1^H NMR spectra of 6 mg/ml Thaumatin samples in HPLC (blue line) and Lieve (orange line) waters **(A)** Amide **(B)** and aliphatic **(C)** regions of ^1^H NMR spectra of 3 mg/ml MNEI samples in HPLC (blue line), Sant’Anna (red line), Rocchetta (green line) and Lieve (orange line) waters.

The low sensitivity of the Thaumatin structure to the environmental conditions (at least within the range explored) reflects also in the ^1^H-NMR spectrum. Indeed, when we dissolved the protein in the two most different types of water, i.e., HPLC and Lieve, despite the pH and ionic strength differences, we obtained 1D spectra completely overlapping. On the other hand, when we performed the same control in the case of MNEI, some differences in the spectra were present, although all the typical signatures of 2D and 3D structure (*i.e.,* the amide protons signal dispersion, the β-sheet diagnostic signals between 5 and 6 ppm and the shielded signal below 0.5 ppm) were well evident. Figure 3 reports the ^1^D NMR spectra of Thaumatin (panel A) and MNEI (panel B and C) respectively.

Our study of water effects on the sweetness of four emblematic sweet proteins confirmed that the ionic content of mineral waters can have a profound influence on sweetness potency. Remarkably, the sweetness trend was confirmed when we analyzed the sweetness of the emblematic proteins, i.e., MNEI and Thaumatin, dissolved in HPLC water in which we had previously added different amounts of NaCl, in order to reproduce the ionic strength of the commercial mineral waters (Supplementary Figure 1). On the other hand, it is known that the mechanism of action of sweet macromolecules can be intrinsically different from that of low molecular weight sweeteners (Temussi et al., 2007). Before drawing possible conclusions on the origin of the “water effect” we performed an experimental control on non-protein sweeteners.

### Sucrose and Sucralose

**FIGURE 4:**
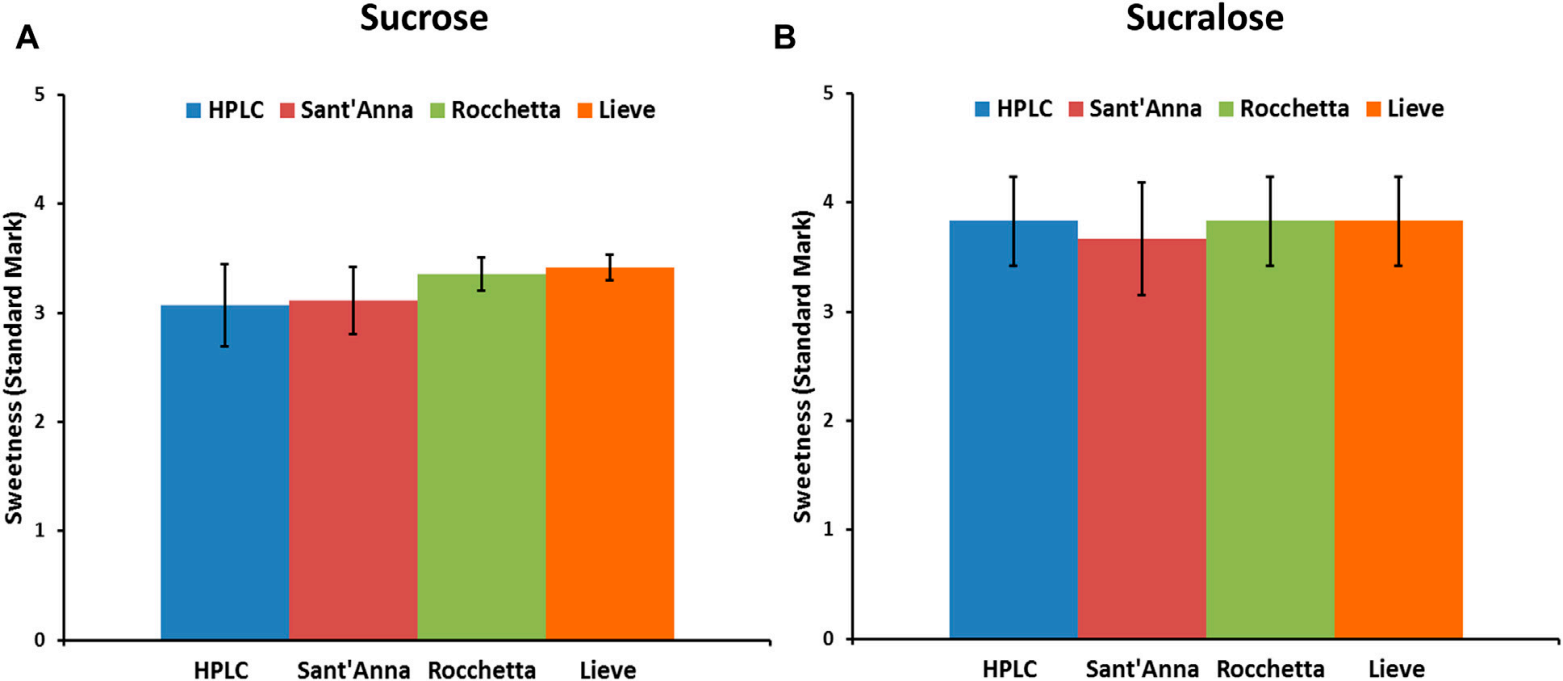
Sensory analysis of low molecular weight sweeteners, sucrose **(A)** and sucralose **(B)** in 4 commercial water types: blue) HPLC, red) Sant’Anna, green) Rocchetta, and orange) Lieve.

Sucrose and sucralose, small molecular weight sweeteners, are commonly consumed sweeteners in different types of foods and beverages worldwide. Herein, we investigated the effect of the same types of commercial waters on the sweetness intensity of sucrose and sucralose. Equal amounts of sucrose (40 g/L) and sucralose (67 mg/L) were used in 4 types of waters to prepare drinking samples for sensory analysis. The concentration of sucrose was selected based on the previous sensory analysis studies (Di Monaco et al., 2014), while the concentration of sucralose was selected according to its sweetness compared to sucrose (600 times sweeter than sucrose). The sweetness intensity of both sweeteners dropped between the levels of sweet and very sweet (Figure 4) and, despite minor differences in the sweetness intensity of sucrose and sucralose in various waters, no correlation with water quality were found.

## Discussion

In the present paper we have described the influence of water quality on the perceived potency of several sweeteners, with a particular emphasis on macromolecular sweeteners. Water quality has no influence on the sweetness potency of low molecular weight sweeteners, i.e., sucrose and sucralose (Figure 4), but it has a marked effect on all sweet proteins explored. Searching for plausible explanations of this behavior we examined the conformation of the sweet proteins with CD and NMR spectroscopies to check conformational changes, and we found that the presence of variable amounts of solid residues in mineral waters has no visible effect on protein conformation (Figures 1–3). These results were not very surprising, since it is unlikely to expect conformational changes induced by a modest increase in ionic strength (Donnarumma et al., 2018). In contrast, we found large differences due to ion content on perceived sweetness of all explored sweet proteins. In fact, the presence of higher amounts of mineral contents resulted in less sweetness power (Figures 1, 2). We then resorted to a detailed analysis of the mechanism of interaction with the receptor.

In the wedge model, it was proposed that, while the equilibrium between inactive and active forms of the receptor is usually shifted by binding small sweeteners to the orthosteric sites, the same shift could be achieved by sweet proteins, which bind like a wedge to an external site of the active form. A crucial aspect of the wedge model is the charge complementarity between the interacting surfaces of the sweet protein and the receptor counterpart (Esposito et al., 2006): all sweet proteins are covered by positively charged residues whereas the external site of the receptor accepting the sweet protein has a prevalence of negative charges. Thus, the mechanism of the macromolecules happens by interaction of two large surfaces with different electrostatic charges.

This accounts for a different behavior for sweet proteins and the low molecular weight ligands, since even a relatively small change in ionic strength of the solution will directly affect the extent of interaction. On the contrary, the interaction of sucrose and sucralose inside the cavity of the orthosteric site is dominated by hydrogen bonds and non-covalent forces the receptor. The influence of the ionic strength of the external solution can only be vanishingly small because the inner walls of the cavity contain only fairly stable water molecules and a few counterions. In conclusion, the different behavior of all explored sweet proteins with respect to the low molecular weight sweeteners reported in this study provides a further experimental evidence, albeit indirect, validating the wedge model which, differently from the previous studies, is based on a functional property.

## Data Availability

The original contributions presented in the study are included in the article/Supplementary Material, further inquiries can be directed to the corresponding authors.
