# Supplementary figures and images for: Striking Dependence of Protein Sweetness on Water Quality: The Role of the Ionic Strength
Source: Front Mol Biosci. 2021 Jul 22;8:705102. doi: 10.3389/fmolb.2021.705102 (PMC8339437; doi:10.3389/fmolb.2021.705102)

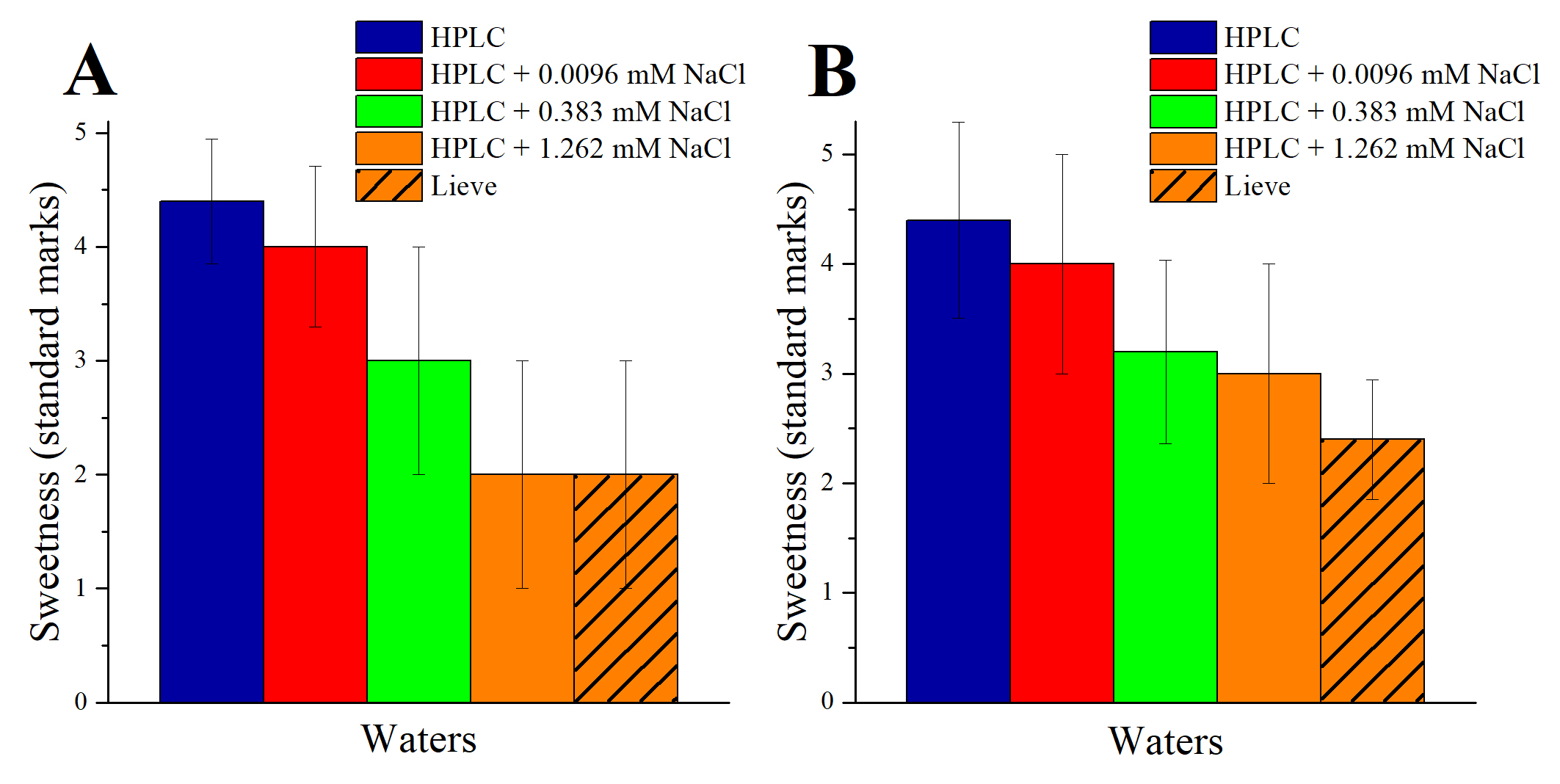

Supplement: Supplementary file 1 [file Image1.jpg]
