# Supplementary material for: Striking Dependence of Protein Sweetness on Water Quality: The Role of the Ionic Strength
Source: Front Mol Biosci. 2021 Jul 22;8:705102. doi: 10.3389/fmolb.2021.705102 (PMC8339437; doi:10.3389/fmolb.2021.705102)
Supplement: Supplementary file 2 [file DataSheet1.docx]

**Figure S1.** Sensory analysis performed on 10 mg/L samples of MNEI (**A**) and Thaumatin (**B**) when dissolved in HPLC water with increasing concentration of NaCl, calculated to match the commercial water conductivity.

**Table S1**. Physicochemical properties including mineral residue amount, conductivity and pH of 4 commercial water types: HPLC, Sant’Anna, Rocchetta, and Lieve. It is also reported the corresponding NaCl concentrations used to simulate the commercial water conductivity.

| Water | Mineral amount (mg/l) | Conductivity (µs/cm) | pH | NaCl concentration (mM) |
| --- | --- | --- | --- | --- |
| HPLC | ≤ 1 | ≤ 1 | 5.90 | n.a. |
| Sant’Anna | 22 | 25 | 7.25 | 0.0096 |
| Rocchetta | 182 | 298 | 7.86 | 0.383 |
| Lieve | 294 | 445 | 7.81 | 1.262 |

**Table S2**. Secondary structure contents of MNEI, Mut3, and Mut9 at 10 mg/L in 4 commercial water types including HPLC, Sant’Anna, Rocchetta, and Lieve by spectral deconvolution using the BestSel online tool (Micsonai et al., 2018). Errors on secondary structure content values are within ± 2%.


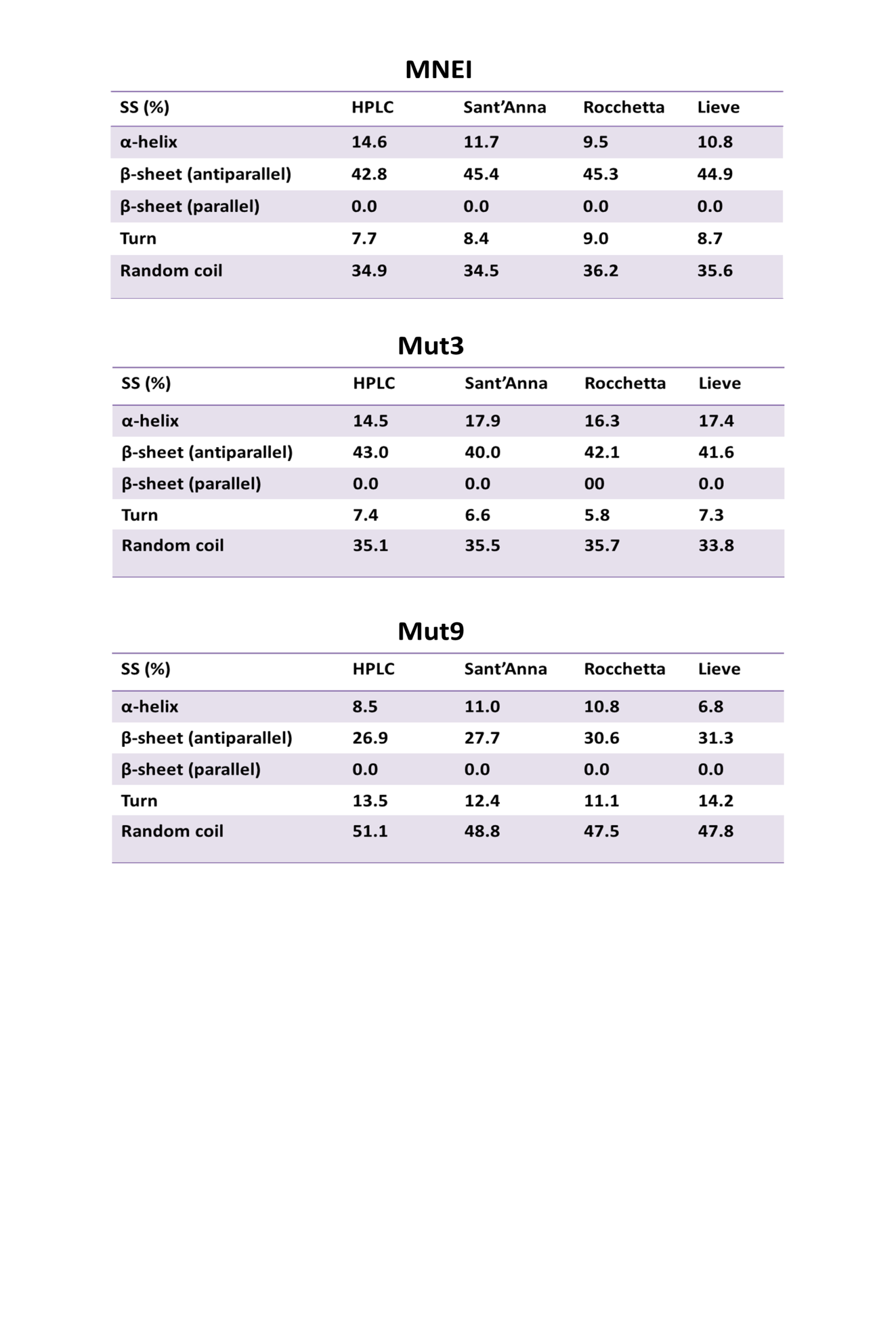


**Table S3**. Secondary structure contents of Thaumatin at 10 mg/L in 4 commercial water types including HPLC, Sant’Anna, Rocchetta, and Lieve by spectral deconvolution using the BestSel online tool (Micsonai et al., 2018). Errors on secondary structure content values are within ± 2%.


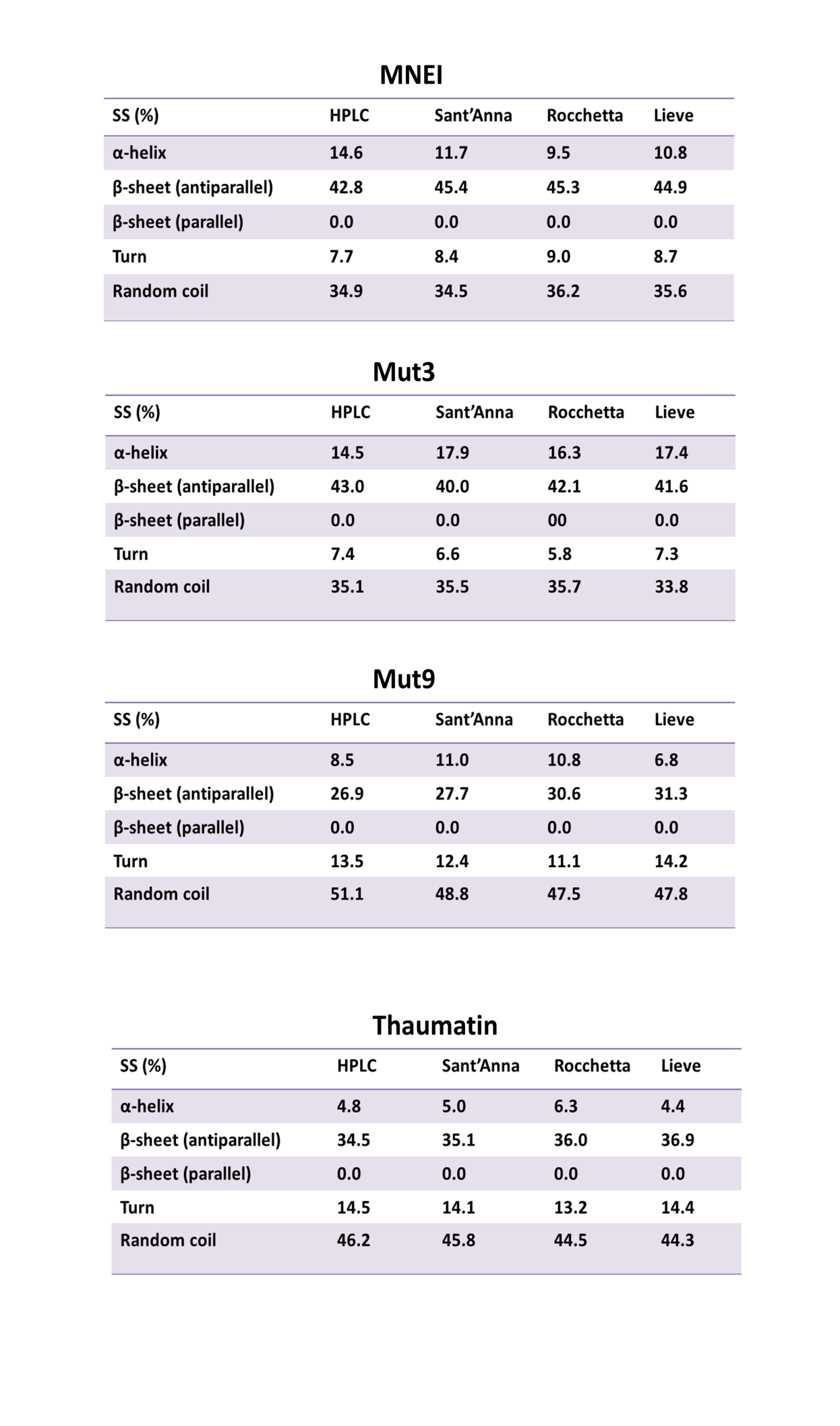


# Reference

Micsonai, A., Wien, F., Bulyáki, É., Kun, J., Moussong, É., Lee, Y.-H., Goto, Y., Réfrégiers, M., & Kardos, J. (2018). BeStSel: a web server for accurate protein secondary structure prediction and fold recognition from the circular dichroism spectra. *Nucleic Acids Research*, *46*(W1), W315–W322.
